# In Vitro Effect of Instrumentation Using Ultrasonication with and without Hydrogen Peroxide on the Removal of Biofilms and Spread of Viable Microorganisms in Aerosols

**DOI:** 10.3290/j.ohpd.b2395059

**Published:** 2022-01-20

**Authors:** Alexandra Stähli, Carla Lanzrein, Egle Milia, Anton Sculean, Sigrun Eick

**Affiliations:** a Senior Dentist and Lecturer, Department of Periodontology, School of Dental Medicine, University of Bern, Bern, Switzerland. Conceptualised the experiments, wrote and proofread the manuscript.; b Dentist in Graduate Program, Department of Periodontology, School of Dental Medicine, University of Bern, Bern, Switzerland. Conducted the experiments, proofread the manuscript.; c Professor, Department of Medicine, Surgery and Experimental Science, University of Sassari, Sassari, Italy. Checked the manuscript and helped with the writing, proofread the manuscript.; d Professor, Department of Periodontology, School of Dental Medicine, University of Bern, Bern, Switzerland. Checked the manuscript and helped with the writing, proofread the manuscript.; e Associate Professor, Department of Periodontology, School of Dental Medicine, University of Bern, Bern, Switzerland. Designed the study, conceptualised the experiments, performed statistical analyses, wrote and proofread the manuscript.

**Keywords:** aerosol, biofilm, hydrogen peroxide, periodontal therapy

## Abstract

**Purpose:**

To evaluate the use of hydrogen peroxide as an adjunct to ultrasonication (US) in biofilm removal and whether it can limit the spread of viable microorganisms in the aerosol.

**Materials and Methods:**

Multi-species biofilms were formed on dentin disks and titanium disks fixed on a plastic surface. After placing the specimens in a periodontal pocket model, an ultrasonic scaler was applied for 30 s, in part combined with 0.25% or 0.5% H_2_O_2_. After treatment, the remaining biofilm was analysed for bacterial counts (colony forming units [CFU]), biofilm quantity and metabolic activity. Further, the cytotoxic effect of hydrogen peroxide on periodontal ligament fibroblasts was assessed and the spread of bacteria in aerosol was quantified.

**Results:**

Ultrasonication reduced bacterial counts in biofilm, biofilm mass and metabolic activity on both dentin and titanium disks. Adjunctive use of 0.25% and 0.5% H_2_O_2_ more effectively reduced the viable bacteria in biofilm than ultrasonication alone; this was also found on both dentin and titanium. The different concentrations of H_2_O_2_ did not lead to corresponding differences in bacterial mass and metabolic activity. The spread of bacteria through aerosols was statistically significantly reduced when adjunctive H_2_O_2_ was used. However, a certain cytotoxic effect on periodontal ligament fibroblasts by H_2_O_2_ could not be ruled out.

**Conclusions:**

Irrigating with H_2_O_2_ during periodontal instrumentation with an ultrasonic scaler increases the reduction of viable bacteria within biofilms. It might limit bacterial spreading via aerosols.

Periodontitis is a chronic inflammatory disease of the tooth supporting tissues associated with high counts of certain bacterial species interacting with the host immune system.^4^ The primary goal in cause-related periodontitis treatment is to remove hard and soft bacterial deposits, which should result in a smooth and biocompatible root surface to facilitate fibroblast attachment and minimise bacterial adhesion.^3^ Despite the fact that hand instruments (i.e. curettes) are still widely used, ultrasonic scalers have gained popularity in dental practice. Comparisons between hand instruments and sonic and ultrasonic scalers did not show a clear advantage for any procedure,^26^ and traumatisation immediately after instrumentation is similar.^2^

Attempts were made to further improve the removal of the biofilms by using an ultrasonic scaler. A recent systematic review evaluated the adjunctive use of antiseptics in clinical trials:^27^ chlorhexidine, essential oils and povidone iodine were used and in part showed beneficial effects.

Over the last 20 to 30 years, dental implants have been widely used to replace missing teeth. The need for prevention and treatment of peri-implant diseases is continually increasing. The prevalence of inflammatory peri-implant diseases is high. In a recent systematic review, the weighted mean prevalence of peri-implant mucositis and peri-implantitis among individuals with implants was 43% and 22%, respectively.^5^

In the 1990s, a low-concentration hydrogen peroxide solution (≤ 3%) was discussed as being beneficial in reducing plaque scores and gingival inflammation.^18^ Hydrogen peroxide (H_2_O_2_) shows oxidising and antiseptic properties. It is effective against a wide range of viruses and bacteria. It exerts its antimicrobial effects by increasing cellular oxidative stress, thereby upregulating the expression of TLR3 and NF-kB, both of which activate the innate local immune response. Hydroxyl radicals that result from H_2_O_2_ cause lethal oxidative damage to microbes, e.g. via DNA oxidation or lipid peroxidation.^23^ For instance, H_2_O_2_ disinfectant proved to inactivate porcine epidemic diarrhea virus in swine feces on metal surfaces,^10^ and at a concentration of 3% was effective against rabies virus.^1^

The outbreak of a novel coronavirus, SARS-CoV-2, in China in December 2019 and its rapid spread raised serious global health concerns. Airborne transmission via respiratory droplets and aerosols, that are abundantly formed during dental treatment, is meanwhile considered the main route of infection.^28^ SARS-CoV-2 infected patients usually carry high loads of the virus in the oropharynx and the oral cavity. Therefore, pretreatment mouthrinses have been proposed to reduce the risk of nosocomial infections by healthcare professionals with close contact to this area, such as dentists or otorhinolaryngologists. One agent that has been proposed in this context is hydrogen peroxide.

A recent review^12^ including 26 studies showed that human coronaviruses such as Severe Acute Respiratory Syndrome (SARS) virus and Middle East Respiratory Syndrome (MERS) virus can persist on inanimate surfaces, but can effectively be inactivated by 0.5% H_2_O_2_ within 1 min.

Therefore, the purpose of the study was to evaluate in an established in vitro model for testing non-surgical therapy: a) the removal of biofilms, b) the potential cytotoxic activity on periodontal ligament cells and c) the spread of viable microorganisms in the aerosol.

The hypothesis was that the adjunctive use of hydrogen peroxide with ultrasonication (US) more effectively removes biofilms and viable microorganisms in aerosol than US alone. The primary outcome is that 0.25% hydrogen peroxide applied for 30 s and left in place thereafter for another 30 s reduces bacterial counts in a 10-species biofilm by about 1 log10 CFU more than US alone.

## Materials and Methods

### Ultrasonic Scaler

The study employed an established in vitro model for testing non-surgical therapy,^8^ using two different surfaces as test specimens (i.e. hydroxyapatite and SLA [sandblasted, large grit, acid-etched] titanium).

In all groups except for the negative control, a piezo scaler (W&H; Bürmoos, Austria) with 2U tips (W&H) for hydroxyapatite surfaces and tip 1I Implant-clean (W&H) for titanium surfaces was used. The exact parameters of the device (angle, distance to the surface, water and power setting) were set according to the manufacturer’s recommendation.

### Biofilm Removal from Dentin and Titanium Specimens

#### Specimens

From a pool of extracted teeth, dentin disks with a diameter of 5 mm were prepared. Before extraction, patients were informed about the use of their teeth for research purposes and their spoken consent was obtained. The present experiment was carried out in accordance with the approved guidelines and regulations of the local ethics committee (KEK) for irreversibly anonymised samples. These dentin disks (diameter 5 mm) and the titanium disks (diameter 5 mm, with SLA surface) were fixed on a plastic surface. In preliminary tests, stable bonding to the plastic surface as the carrier was established.

#### Biofilm formation

All disks were colonised with a biofilm. For biofilm formation, a multiple species mixture consisting of 10 bacterial strains (*Streptococcus gordonii* ATCC 10558, *Actinomyces naeslundii* ATCC 12104, *Fusobacterium nucleatum* ATCC 25586, *Campylobacter rectus* ATCC 33238, *Eikenella corrodens* ATCC 23834, *Parvimonas micra* ATCC 33270, *Prevotella intermedia* ATCC 25611, *Porphyromonas gingivalis* ATCC 33277, *Tannerella forsythia* ATCC 43037, *Treponema denticola* ATCC 35405) was prepared. Before an experiment, all strains (except for *T. denticola* ATCC 35405) were precultivated on Schaedler agar plates (Oxoid; Basingstoke, UK) with 5% sheep blood in an anaerobic atmosphere or with 5% CO_2_ (*S. gordonii* ATCC 10558). *T. denticola* ATCC 35405 was anaerobically cultured in modified mycoplasma broth (BD; Franklin Lakes, NJ, USA) to which 1 mg/ml glucose, 400 µg/ml niacinamide, 150 µg/ml spermine tetrahydrochloride, 20 µg/ml Na isobutyrate enriched with 1 g/ml cysteine and 5 µg/ml cocaboxylase had been added.

First, 1.5% bovine serum albumin (BSA) solution was added to the disks for 10 min before placing them in tubes. Then the bacterial suspension was added. The medium was first changed after 48 h. *P. gingivalis* ATCC 33277, *T. forsythia* ATCC 43037 and *T. denticola* ATCC 35405 were again added to the nutrient medium before application on the disks. The renewed addition of selected bacterial strains guaranteed a sufficient number of these species in the biofilms.

### Treatment

Treatment of specimens started after 3.5 days of biofilm formation. The disks were placed in the periodontal pocket model.^8^

The treatment protocol included two different modalities (1 and 2) and each with one treated control (US only, 3.) and one untreated control (4).

- Specimens were treated with US combined with 0.25% H_2_O_2_ for 30 s
- Specimens were treated with US combined with 0.5% H_2_O_2_ for 30 s
- Specimens were treated with US only for 30 s
- Specimens were left untreated.

All treatments were performed by the same calibrated operator (CL). After treatment, the remaining biofilm was analysed.

### Analysis of Biofilm

At least six independent results were achieved for each type of treatment, disk surface and experiment.

On colonised and treated disks, biofilm samples were scraped from the surface and suspended in 0.9% NaCl solution. After making a serial dilution of each, 25 µl were spread on Schaedler agar plates, incubated under anaerobic conditions, and the total CFU were counted and recorded. The biofilms were quantified according to recently published protocols.^16^ After rinsing and heat-fixing the biofilms at 60°C, biofilms were stained with 50 µl of 0.06% (w/v) crystal violet (Sigma-Aldrich; St Louis, MO, USA) per well for 10 min. The staining was quantified using a plate reader (ELx808, Biotek Instruments; Winooski, VT, USA) at 600 nm.

### Biofilm Metabolic Activity

Biofilm metabolic activity was assessed using Alamar blue as a redox indicator.^22^ Five µl of Alamar blue (alamarBlue reagent, Thermo Fisher Scientific; Waltham, MA, USA) was mixed with 100 µl of the nutrient medium and added to the biofilm. After extensive mixing with the biofilm and an incubation for 1 h at 37°C, absorbance was measured at 570 nm against 600 nm by using a microplate reader (ELx808, Biotek).

Experiments were made each in triplicates in three independent series resulting in at least nine single values each.

### Cytotoxic Effect of Treatment on Periodontal Ligament Fibroblasts

Human periodontal ligament fibroblasts were anonymously collected from patients during regular surgical treatment (wisdom tooth removal) following written informed consent. This did not require approval by the Ethics Committee of the University of Bern. Fibroblasts were placed in T-25 cell culture flasks containing DMEM (Life Technologies / Invitrogen; Paisley, UK) with 10% foetal calf serum (FCS; Life Technologies / Invitrogen) to grow to confluency. At the beginning of the experiments, the fibroblasts were always in the 4th–6th passage.

Monolayers of fibroblasts were exposed to hydrogen peroxide solutions in different concentrations (two-fold dilution series, starting from 0.5% down to 0.008%). After 30 s, the hydrogen peroxide solution was replaced by cell cultivation medium and cells were cultivated for an additional 6 h before cytotoxicity against cells was determined by MTT assay according to Mosmann.^20^ Experiments were made in independent sextuplicates.

### Spreading of Aerosols

Biofilms were formed in 96-well-plates as described before. Nutrient broth was removed. Then the tip of the ultrasonic device was placed close to the bottom and the biofilm was treated for 30 s. Agar plates (diameter 9 cm) were placed at a distance of 5 cm. After the treatment, the plates were incubated anaerobically.

### Statistical Analysis

ANOVA with a post-hoc Bonferroni test was used for statistical analysis. The level of significance was set at p = 0.05. SPSS 24.0 software (IBM; Armonk, NY, USA) was used.

## Results

### Biofilm Reduction

**Fig 1:**
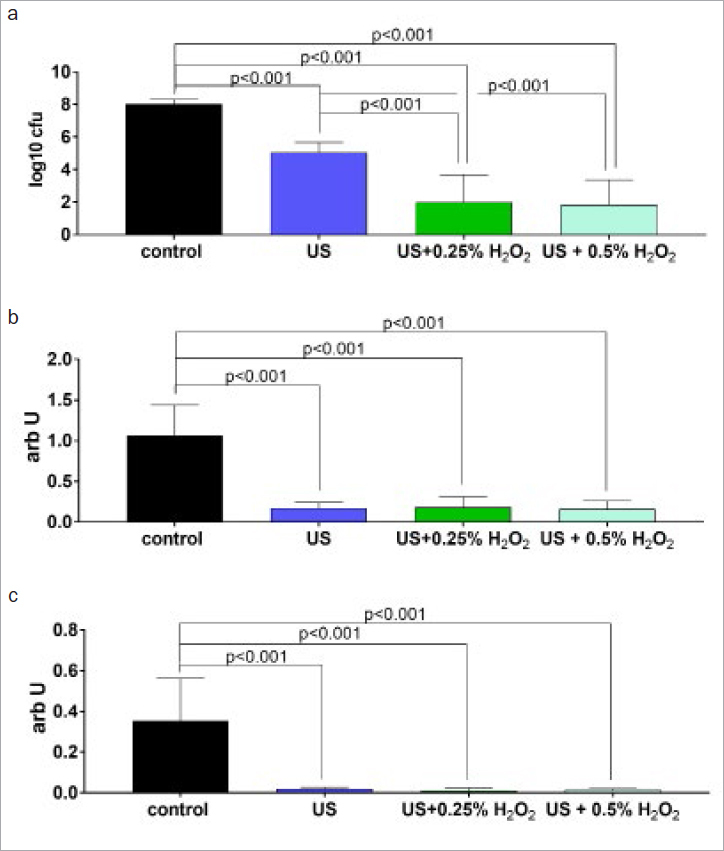
Mean (and SD) of total counts of colony forming units (CFU; a), mass (b) and metabolic activity in biofilm on dentin disks after 30 s of instrumentation with an ultrasonic scaler (US) without and with 0.25% and 0.5% H_2_O_2_.

**Fig 2:**
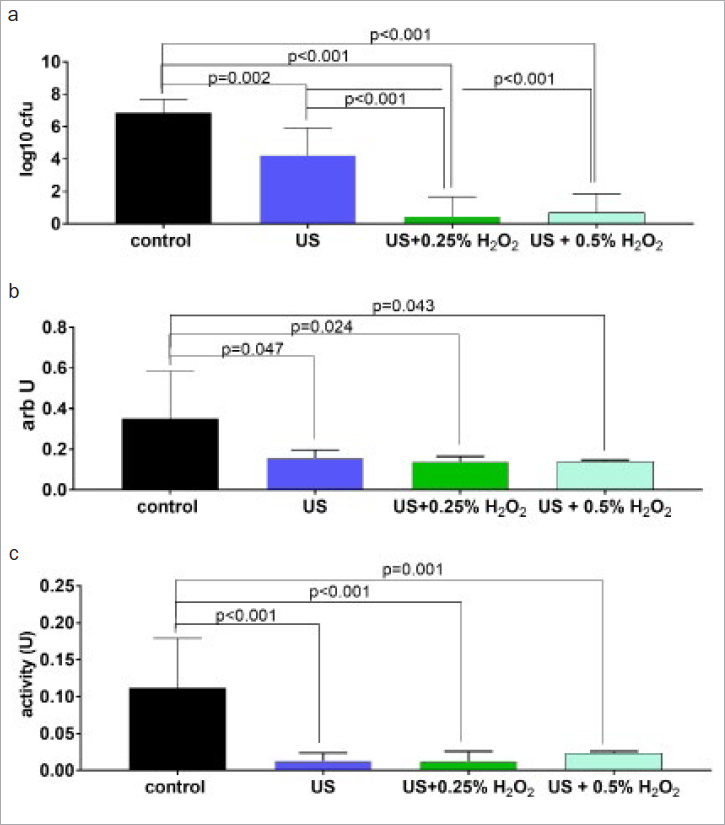
Mean (and SD) of total counts of colony forming units (CFU; A), mass and metabolic activity in biofilm on titanium disks after 30 s of instrumentation with an ultrasonic scaler (US) without and with 0.25% and 0.5% H_2_O_2_.

Ultrasonication with water as a cooling medium decreased bacterial counts in biofilm by 3 log10 CFU (dentin) and 2.60 log10 CFU (titanium), and it also reduced biofim mass and metabolic activity. Ultrasonication of dentin disks with both 0.25% and 0.5% H_2_O_2_ statistically significantly (p < 0.001) reduced the bacteria in the biofilm compared to US alone (Fig 1). Similar results were observed for titanium disks. Both concentrations statistically significantly reduced bacterial counts compared to US alone (Fig 2). With regard to bacterial mass and metabolic activity, no differences between H_2_O_2_ concentrations were detected (Figs 1 and 2).

### Spreading of Aerosols

Instrumentation in the periodontal pocket was always performed with an efficaceous evaporation system. The first-performed analyses in the surroundings resulted in only negative results. Subsequently, the protocol was repeated without evaporation, and the results (Table 1) showed a reduction of viable bacteria following the adjunctive use of H_2_O_2_ as indicated by the numbers of overall positive samples and the mean counts of positive samples.

**Table 1:** Bacterial CFU per 63.5 cm^2^ in agar plates at a distance of 5 cm from the ultrasonic scaler application site

	Positive samples	Positive samples (%)	Mean count (CFU) of positive samples
US	4/4	100	17.75
US + 0.25% H_2_O_2_	2/4	50	7.5
US + 0.5% H_2_O_2_	1/4	25	0.5

### Vitality of Periodontal Ligament Fibroblasts

**Fig 3:**
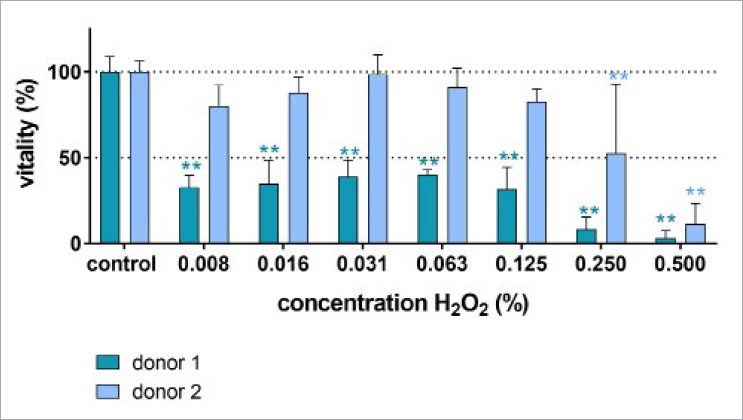
MTT ((3-(4,5-dimethylthiazol-2-yl)- 2,5-diphenyltetrazolium bromide) tetrazolium) assay assessing the vitality of the periodontal ligament fibroblasts after exposure to different concentrations of hydrogen peroxide for 30 s and subsequent cultivation for 6 h. ** p<0.01 vs the unexposed control of the respective donor.

In the assays, periodontal ligament fibroblasts of two donors were used. Whereas cells of donor 2 lost vitality only when exposed to 0.25% and to 0.5% hydrogen peroxide, the cells obtained from donor 1 showed less vitality after all concentrations of hydrogen peroxide tested (Fig 3).

## Discussion

This research was prompted not only by the intention to disinfect periodontal or peri-implant pockets concomitant with mechanical debridement, but also by the current global health concern about the SARS-CoV-2 pandemic and the ongoing discussion on aerosol contamination. The objective of the present in vitro study was to investigate whether the use of H_2_O_2_ solution adjunctive to an ultrasonic scaler augments the antibiofilm effect when used on dentin and titanium surfaces and if it was bactericidally active in the generated aerosols.

Our results demonstrated that H_2_O_2_ is able to reduce bacterial counts in biofilms on both dentin and titanium surfaces after instrumentation. However, H_2_O_2_ could not statistically significantly reduce the metabolic activity of bacteria within the remaining biofilm on both dentin and titanium surfaces. Furthermore, H_2_O_2_ showed the potential to reduce the spreading of bacteria via aerosols that are generated during mechanical debridement of inflamed periodontal or peri-implant pockets. Thus, this property might also be relevant for reducing viral transmission through aerosols.

H_2_O_2_ plays an important role in biofilm homeostasis. It is the major substance synthesised by commensal oral species to inhibit growth of pathogens.^9^ Oral streptococci represent the predominant species which are known to produce H_2_O_2_.^14^ Among them, *Streptococcus sanguinis, Streptococcus gordonii*, and *Streptococcus oralis* show inhibitory effects on the growth of *A. actinomycetemcomitans, P. gingivalis* and *P. intermedia*. Environmental alterations, however, can substantially influence or even neutralise this inhibitory effect of H_2_O_2_ and shift the equilibrium within the biofilm towards dysbiosis.

Previous research demonstrated that hydroxyl radicals generated from H_2_O_2_ by photocatalysis and applied after non-surgical therapy with US were more effective in reducing pocket probing depths and improving clinical attachment levels than US alone or US plus locally delivered minocycline gel.^13^ It can be speculated that these results might be a consequence of the bactericidal effect of H_2_O_2_ at the bottom of the periodontal pocket, creating an environment facilitating the healing process. The instability of hydrogen peroxide limits its use as a therapeutic agent; hydroxyl radicals generated by H_2_O_2_ have a very short half-life of approximately 10^-9^ s.^11,25^ Several attempts have been made to overcome this problem, e.g. silver was added to stabilise the molecule.^19^

In this study, we assessed bacterial counts in biofilms after treatment with US, US+0.25% H_2_O_2_ and US+0.5% H_2_O_2_. We found a statistically significantly stronger inhibitory effect when adjunctive H_2_O_2_ was used. Interestingly, no statistically significant difference between the two concentrations used was observed. These positive effects of H_2_O_2_ are in keeping with the findings of previous studies that reported similar effects when *Staphylococcus aureus* suspensions were exposed to H_2_O_2_ together with laser light and ultrasound, while ultrasonic irrigation alone had no effect.^23^ Moreover, in terms of oxidative stress, the combination of H_2_O_2_, laser light and US was most effective in inducing oxidative damage of bacterial DNA.^23^ A recent study treated titanium surfaces colonised by biofilms characteristic of peri-implantitis with an antimicrobial therapy employing H_2_O_2_ photolysis.^11,21^ Similar to our experimental setting, titanium disks colonised by a 3-species biofilm were subjected to ultrasonic scaling and application of 3% H_2_O_2_ photolysis. The results are in agreement with those of the present study, despite the differences in biofilm composition and H_2_O_2_ concentration (i.e. ultrasound scaling eliminated large portions of the biofilm by 3 log10, while additional treatment with H_2_O_2_ resulted in no detectable viable bacteria).^21^

Subsequently, we attempted to determine the biofilm mass and metabolic activity of the biofilm. All biofilms treated with US revealed a statistically significant reduction compared to the untreated control. However, no statistically significant differences among the groups were observed, pointing to the obvious effects exerted by US.

Next we looked at the spreading of bacteria via aerosols. A first round of experiments was performed using an effective evaporation system, while the next series was done without. While no spreading was observed when evaporation was used, ultrasonication alone without evaporation resulted in a conspicuous spreading of bacteria with 100% infected wells at a distance of 5 cm. Interestingly, H_2_O_2_ statistically significantly reduced the spreading effect. Clinically, these results indicate that 1. suction during mechanical therapy might be of importance to reduce bacterial loads of aerosols and 2. that H_2_O_2_ irrigation might further prevent bacterial spreading. In this context, a prospective clinical trial investigated the viral load of hospitalised SARS-CoV-2 patients before and after mouthrinsing with 1% H_2_O_2_ and found no statistically significant change in the oral viral load.^6^ However, the viral load was tested on RNA level, and these results indicate that H_2_O_2_ may not be able to inactivate RNA even though the viral cells might have been destroyed.

Another important aspect that needs to be discussed is the fact that bactericidal effects largely depend on whether bacteria are planktonic or in established biofilms. For instance, antibacterial photodynamic therapy (aPDT) shows substantially less activity on established biofilms than on planktonic periodontopathogenic bacteria.^15^ To increase the activity also against an established biofilm, an adjunctive irrigation with H_2_O_2_ might exert a synergistic effect. This question was investigated in a previous study by our group, also using the pocket model.^15^ While biofilms treated mechanically or with aPDT were reduced by less than 0.5 log10, the use of 3% H_2_O_2_ after mechanical therapy or aPDT completely eradicated the bacteria. Without mechanical therapy or aPDT and for biofilms cultivated in well plates, 3% H_2_O_2_ caused a reduction by 4 log10.^15^ The bactericidal potential of H_2_O_2_ is further bolstered by its effect on antibiotic-resistant bacteria, another emerging major threat to global health. H_2_O_2_ in combination with UV irradiation enhanced killing of methicillin-resistant *Staphylococcus aureus* (MRSA) and multi-drug resistant *Pseudomonas aeruginosa*.^7^

For the present study, we used the aforementioned periodontal pocket model. Further, in order to better mimic the periodontal pocket with its complex biofilm structure, all specimens were incubated with a multiple-species mixture consisting of 10 bacterial strains. Then, the specimens were inserted into the pocket model. Nevertheless, this model has some limitations, as the cellular interactions and cytokine secretion of epithelial cells, periodontal ligament cells, fibroblasts, macrophages and other cells stemming from the hematopoietic lineage are lacking. Neither does the oxygen content or pH reflect the in vivo situation. Thus, due to these missing manifold interactions, it is very difficult to extrapolate the results obtained in the present study to the in vivo situation. It cannot be ruled out that in a real clinical situation, it is more challenging to reach difficult anatomical areas/site of inflammation, such as furcations or deep pockets, with H_2_O_2_ irrigation.

Finally, we also attempted to assess the cytotoxicity of H_2_O_2_, which exhibited some cytotoxic activity on periodontal ligament fibroblasts. This cytotoxic activity was dependent on the concentration of H_2_O_2_, but there were clear differences in the reaction of the cells used. As those were anonymously collected, no further information on the donors is available. From other experiments in different studies, heretofore unpublished, it is of interest to note that cells showing less cytotoxic reaction produced much less MMP1 than cells with greater cytotoxic reaction, which responded with increased production. A cytotoxicity of 1% H_2_O_2_ against human gingival fibroblasts but also against human epithelial HSC-2 and murine osteoblastic MC3T3-E1 cells has been reported elsewhere.^24^ However, it should also be mentioned that in comparison to hydrogen peroxide, the widely used antiseptic chlorhexidine is already cytotoxic at much lower concentrations.^17^

## Conclusion

The present results indicate that irrigating with H_2_O_2_ during periodontal instrumentation by means of ultrasonication increases the reduction of viable bacteria within biofilms and may limit bacterial spreading via aerosols, thus reducing the risk of bacterial transmission.
